# Comparison of Gut Microbiological Profiles of Inbred and Outbred Healthy Mice

**DOI:** 10.1002/mbo3.70134

**Published:** 2025-11-20

**Authors:** Xudong Liu, Shitao Lian, Aoyi Xiao, Xiafei Hong, Dingyan Cao, Xinjie Xu, Yanan Shi, Qing Zhong, Hangqi Liu, Wenjing Wang, Jinyuan Wang, Zilong He, Wenming Wu

**Affiliations:** ^1^ Laboratory Animal Research Facility, National Infrastructures for Translational Medicine, Institute of Clinical Medicine, Peking Union Medical College Hospital Chinese Academy of Medical Science and Peking Union Medical College Beijing China; ^2^ The State Key Laboratory for Complex, Severe, and Rare Diseases Peking Union Medical College Hospital Beijing China; ^3^ School of Engineering Medicine Beihang University Beijing China; ^4^ Beijing Advanced Innovation Center for Big Data‐Based Precision Medicine, Interdisciplinary Innovation Institute of Medicine and Engineering Beihang University Beijing China; ^5^ Department of General Surgery, Peking Union Medical College Hospital Chinese Academy of Medical Science & Peking Union Medical College Beijing China; ^6^ Biomedical Engineering Facility of National Infrastructures for Translational Medicine, Institute of Clinical Medicine, Peking Union Medical College Hospital Chinese Academy of Medical Sciences and Peking Union Medical College Beijing China; ^7^ Department of Pathology, Molecular Pathology Research Center, Peking Union Medical College Hospital Chinese Academy of Medical Sciences and Peking Union Medical College Beijing China

**Keywords:** 16S V3‐V4, gut microbiota, inbred, mice, outbred

## Abstract

Gut microbes are closely related to host immunity and health, and mice are frequently used as a common model organism in biomedicine to study various diseases. Numerous studies based on mouse gut microbes have been conducted, but whether there are differences in the gut microbial profiles of healthy mice of different strains has not been revealed. In this study, we performed a meta‐analysis comparing four strains (inbred strains: C57BL and BALB; outbred strains: KM and ICR) of mouse healthy gut microbial 16S V3‐V4 data based on publicly available online data. We focused on microbial diversity, microbial composition, abundance differential microbiota, and co‐abundance networks. We found that the gut microbes of these four strains of mice differed in the above metrics to varying degrees. Our study found significant differences in gut microbiology among four strains of healthy mice. The strain will be a background factor that cannot be ignored in future studies of gut microbiology in mice. The impact of this factor on gut microbiology experiments should be considered.

## Graphical abstract

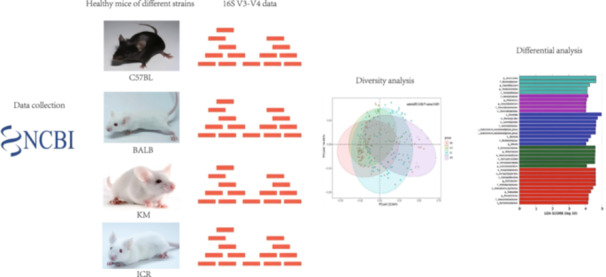

## 1 Introduction

Gut microbiota are intricately associated with host health (Marchesi et al. 2016), and numerous studies have reported significant correlations between gut microbes and various diseases, including intestinal cancer (JOBIN 2017), diabetes mellitus (Qin et al. 2012), cancer (Schwabe and Jobin 2013), and cardiovascular diseases (Tang and Hazen 2014). As the most common experimental animal in basic medicine, mice make important contributions to human disease research. The mouse intestine is frequently employed in research. By collecting mouse faeces or different intestinal segments of the intestine, researchers utilize high‐throughput sequencing to investigate the interaction between gut microbiota and their hosts. In addition to high‐throughput sequencing, researchers have also studied the effects of changes in the gut microbial community on the host in different strains of mice through colony fixation, faecal transplants, and germ‐free mice (Arrieta et al. 2016).

Currently, several mouse strains are commonly employed in basic medical research, including C57BL, BALB, KM, and ICR. Among them, C57BL and BALB are inbred strains of mice, which have a common genetic origin, lack crossbreeding with foreign breeds, are highly genetically pure and have stable characteristics. C57BL (Cheng et al. 2004; Simon et al. 2013) is the most frequently used inbred strain of mice, noted for its long lifespan, ease of maintenance, and propensity for obesity. It is mainly used in cardiovascular, diabetes, obesity, and neurological research. BALB mice (Potter 1985), derived from house mice, are docile, easy to breed, and susceptible to pneumonia and spontaneous hypertension, making them commonly used in oncology, tumor, and hypertension research. KM mice (Yu et al. 2014) are the most widely produced outbred mice in China, possessing a large gene pool, high genetic heterozygosity, low spontaneous tumor rate, and strong disease resistance. They are extensively used in pharmacology and toxicology studies. ICR mice (Cui et al. 1993; Chia et al. 2005; Lehoczky et al. 2006; Brown et al. 2000) are adaptable, robust, and exhibit good experimental reproducibility, making them suitable for haematology, pharmacology, toxicology, and oncology research, similar to KM mice. Although these mice are involved in gut microbiota studies to varying degrees, their genetic background differences lead to variations in their intestinal microbiota. Up to now, a systematic comparison of the intestinal microbes of different strains in mice remains to be further explored and established.

In this study, we collected data on 260 cases of mouse intestinal microbiota (two inbred strains: C57BL and BALB, and two outbred strains: KM and ICR) from publicly available databases for meta‐analysis. We only selected the 16S V3‐V4 compartments data for the systematic study. Our objective was to compare the differences and similarities in microbial community composition both between and within different strains through a meta‐analysis of 16S data, aiming to minimize errors caused by variations in their gut microbiota.

## 2 Methods

### 2.1 Data Download and Screening

To explore the literature highly relevant to mouse gut microbiology, we conducted a comprehensive exploration of the PubMed database. Immediately afterwards, we carried out a meticulous screening of the retrieved literature and ensured that only those samples containing meta‐information and related to healthy mice were retained. Subsequently, by leveraging the existing resources, we downloaded data from public databases. These data originated from the sequencing results of samples taken from four healthy mouse strains, namely C57BL, BALB, KM, and ICR, using the 16S V3‐V4 region, and would serve as the basis for our subsequent analysis. Through extensive literature review, we learned that before the experiment was carried out, these mice had different origins. However, they all came from experimental animal suppliers with professional qualifications and a good reputation. These suppliers strictly adhered to industry norms and ethical guidelines and adopted a scientific breeding and management system during the mouse breeding process to minimize the potential adverse impact of individual differences among mice on experimental results, thus providing a reliable and accurate data foundation and strong experimental support for subsequent scientific research. They were all housed in specific pathogen‐free (SPF) animal facilities. These facilities strictly followed the SPF animal housing standards and provided a stable and suitable living environment for the mice. The mice were kept under extremely precise standard conditions, with the ambient temperature always maintained within the range of 22* ±* 2°C, the air humidity precisely controlled between 40% and 70% and a 12/12‐h light/dark cycle followed to simulate the natural circadian rhythm, ensuring that the mice's biological clocks were not disrupted.

### 2.2 Sequencing Data Processing and Taxonomy Classification

Raw sequencing data were downloaded from the NCBI database (https://www.ncbi.nlm.nih.gov/sra). The raw data were preprocessed using Trim galore (https://github.com/FelixKrueger/TrimGalore) to remove adapter sequences and low‐quality bases. For 16S data, we used QIIME2 (Bolyen et al. 2019) (https://qiime2.org/) to analyse the data. The raw data were denoised into amplicon sequence variation tables (ASVs) using DADA2 (Callahan et al. 2016). Taxonomy classification was performed according to version 138.1 of the Silva database (Robeson et al. 2020; Quast et al. 2012). For subsequent analyses we retained relative abundance results at the phylum and genus level for taxonomic information. We assessed the relationship between microbial composition in each group using the Bray‐Curtis distance to visualize subject separation based on pairwise distances. Principal coordinate analysis (PCoA) plots were generated using the first two principal coordinates and labeled according to each mouse strain. We reflected the abundance and diversity of microbial communities by the Evenness and Shannon Index. We used the Rarefaction Curve to compare species richness in samples with different amounts of sequencing data. The Shannon‐Wiener index reflects the diversity of microorganisms in the samples, and we constructed curves using the microbial diversity index of each sample at different sequencing depths to reflect the microbial diversity of each sample at different sequencing quantities.

### 2.3 Identification of Abundance‐Differentiated Microbiota and Clustering Analysis

We used LEfSe (Segata et al. 2011) (https://github.com/SegataLab/lefse) to separately analyse the differential microbiota of the different strains in mice at the genus level from 260 cases. This analysis first employed a non‐parametric Kruskal‐wallis rank sum to detect species with significant differences in abundance between subgroups, to find taxa with significant differences in abundance, and then used linear regression analysis (LDA) to estimate the magnitude of the effect of abundance on the differential effect of each species, to obtain the linear regression scores of the different species, and to identify species with significant differences (LDA > 3). At the same time, we also performed hierarchical clustering within and between mouse strains based on species abundance by the hclust algorithm.

### 2.4 Construction of Co‐Abundance Network of Gut Microorganisms in Different Strains of Mice

We used the Hmisc package (https://github.com/cran/Hmisc) to calculate Spearman rank correlations (*r* > 0.4 and *p* < 0.05) between genus‐level ASVs. Network topological properties including number of nodes, number of edges and average degree were calculated using Cytoscape (https://cytoscape.org/download.html) and a co‐occurrence network graph was drawn based on the network properties.

### 2.5 Data Statistics and Visualization

All processed data, if not otherwise stated, were loaded in the R language environment (version 4.3.2 https://www.r-project.org/) and analyzed and visualized. Comparisons between the two groups were made using the wilcox test, and correlation analyses were performed using the Spearman correlation test. All tests of significance were two‐sided, with *p*‐values < 0.05 (two‐group comparisons) or corrected *p*‐values < 0.05 (multiple comparisons) considered statistically significant.

## 3 Result

### 3.1 Collation of Gut Microbiological Data and Diversity Analysis of Different Strains of Mice

**Figure 1:**
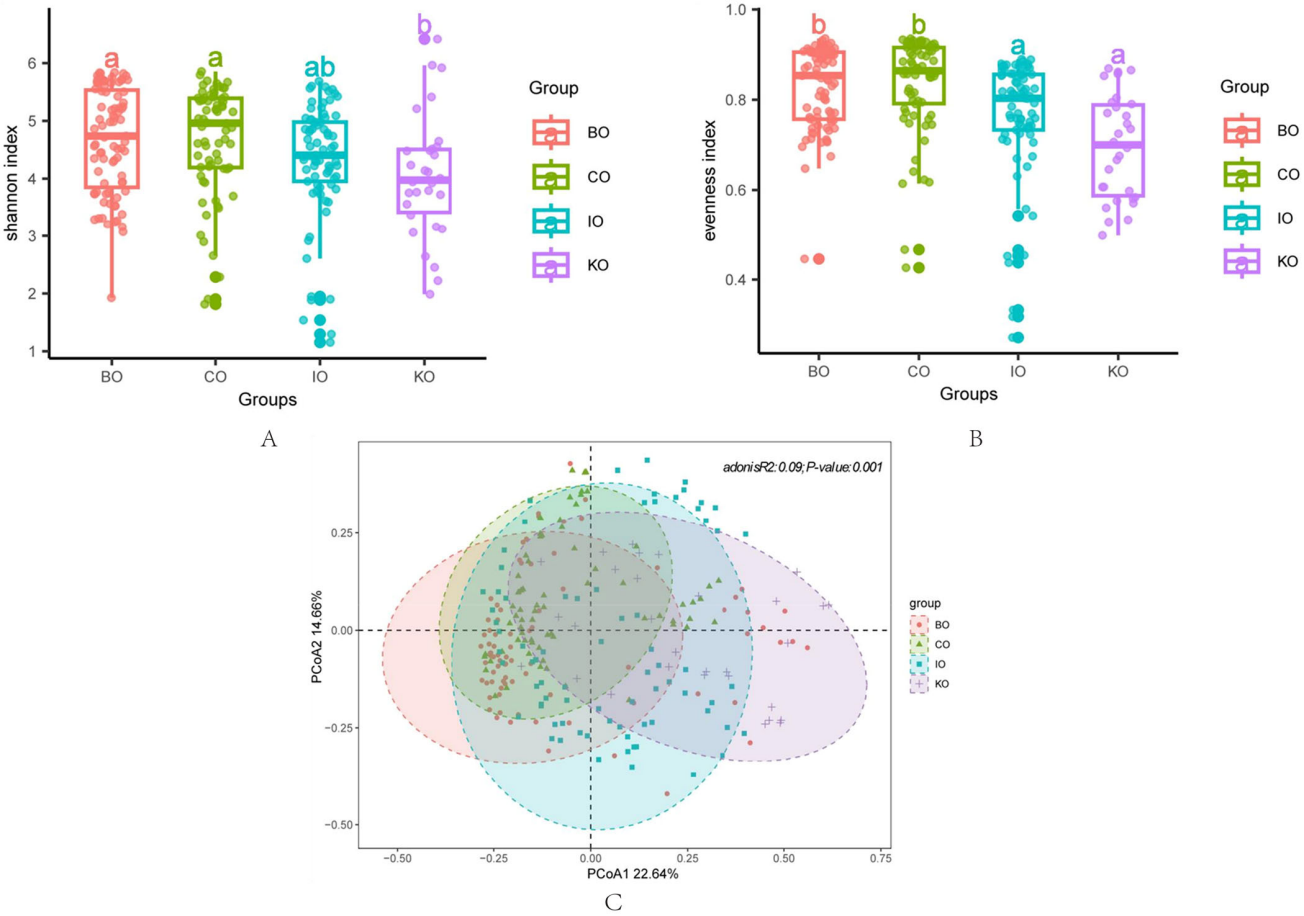
(A) Gut microbial Shannon index distribution of four strains mice. (B) Gut microbial Evenness index distribution of four strains mice. Figure 1A and 1B Present the distribution of Shannon and Evenness indices for gut microbiota in four groups of mice (BO, CO, IO, KO). The groups are distinguished by different colors in the figures, and the letters annotated on the scatter plots indicate whether there are significant differences between groups. Different letters signify significant differences between groups (*p* < 0.05, as determined by ANOVA and Tukey‐HSD tests), allowing for the comparison of gut microbiota diversity among the four groups of mice. (C) PCoA analysis of four strains mice. Figure 1C Shows the results of PCoA based on Bray‐Curtis dissimilarity, where each point represents an individual sample, and points of different colors correspond to different groups (e.g., BO, CO, IO, KO). PERMANOVA is used to analyze the degree of explanation of sample differences by different grouping factors, and permutation tests are used for significance statistics.

We collected gut microbiological data from four different strains of mice from multiple studies with a total of 260 samples. These samples consisted mainly of two inbred strains (C57BL abbreviation: CO, *n* = 70 and BALB abbreviation: BO, *n* = 83) and two outbred strains (KM abbreviation: KO, *n* = 30 and ICR abbreviation: IO, *n* = 77) (Supporting Information S4: Table S1). To ensure comparability of the data, only the 16S V3‐V4 samples were selected for subsequent analyses. With regard to α‐diversity, among the four groups, the CO group exhibited the highest median Shannon index, followed by the BO group, while the KO group had the lowest median. Both CO and BO did not significantly differ from each other but were significantly different from KO (Figure 1A). A similar trend was observed for the Evenness index, with higher medians for CO and BO, and lower medians for IO and KO, indicating significant differences between inbred and outbred lines (Figure 1B). Regarding β‐diversity, the samples of IO exhibited greater divergence from other groups, while the KO sample distribution was less pronounced, likely due to the smaller sample size. The BO and CO sample distributions were more closely aligned (Figure 1C).

### 3.2 Intestinal Bacteria Taxonomy Classification of Different Strains of Mice

**Figure 2:**
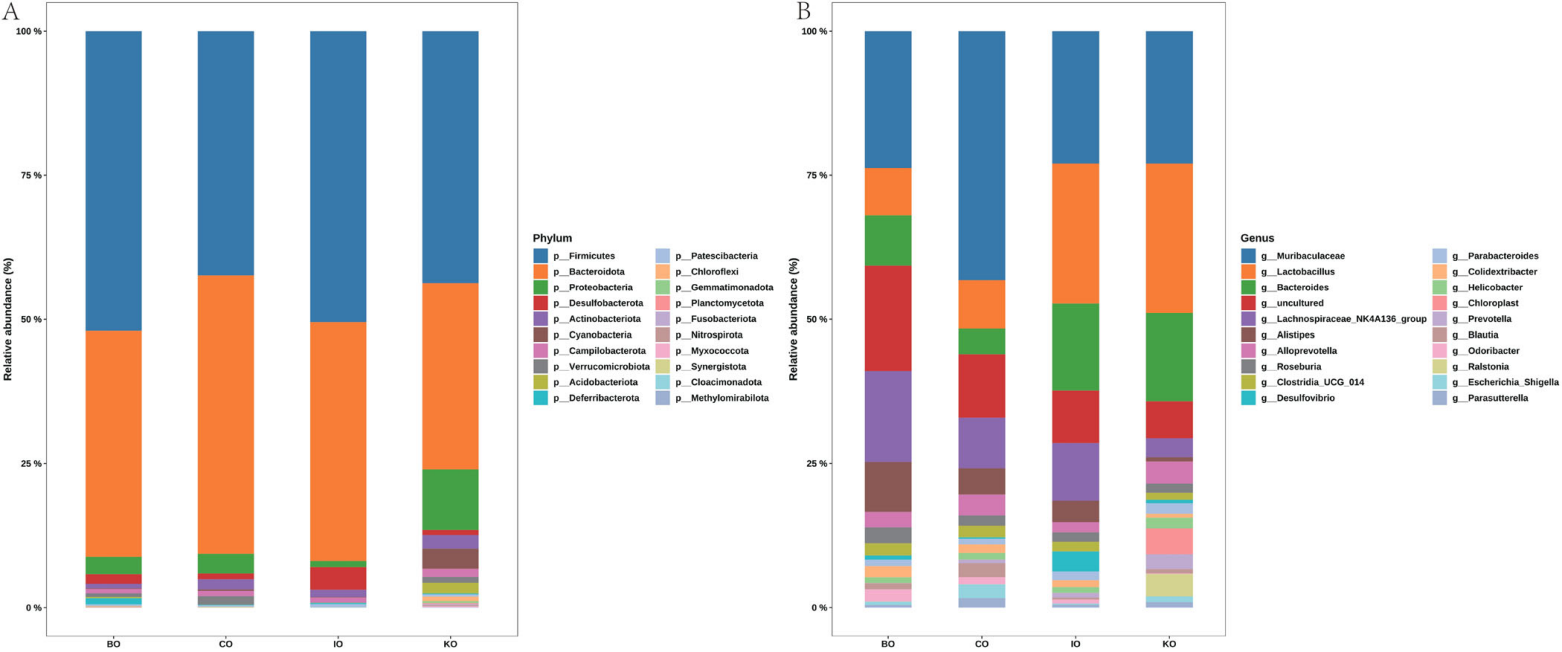
(A) Gut microbial composition of four strains mice at phylum level. (B) Gut microbial composition of four strains mice at genus level. Figure 2 clearly shows the relative abundance of the gut microbiota in four groups of mice, namely the BO group, the CO group, the IO group and the KO group. The gut microbiota are classified according to the classification criteria of Phylum and Genus. Here, the relative abundance is intuitively presented in the form of percentages, and each color precisely corresponds to a specific taxonomic group at the Phylum or Genus level.

**Figure 3:**
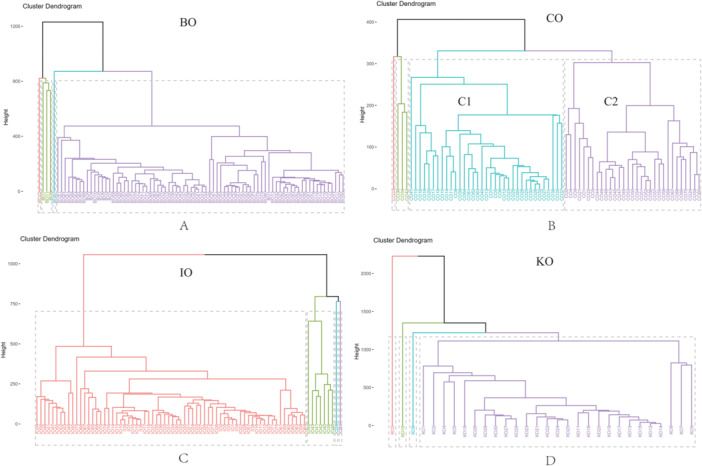
(A) Hierarchical clustering of BO mouse strains based on gut microbial abundance. (B) Hierarchical clustering of CO mouse strains based on gut microbial abundance. (C) Hierarchical clustering of IO mouse strains based on gut microbial abundance. (D) Hierarchical clustering of KO mouse strains based on gut microbial abundance. In the clustering analysis process of Figure 3, dendrograms are generated by calculating the distance metrics between data points. The “height” on the Y‐axis represents the distance metric when two clusters are merged during the clustering process. Different colors are used in the figure to represent different clusters. The red and green branches in Figure 3 (A) (IO group) represent different clusters. Dashed lines are used to indicate the cut‐off points or thresholds of clustering, which can determine how data points are divided into different clusters at a specific similarity level. The use of colors and dashed lines helps to intuitively understand the clustering structure and similarity of data points in different groups.

We further analyzed the taxonomy classification of the gut microbes across the four mouse strains. At the phylum level, the primary mouse gut microbes were similar across the strains, mainly dominated by *Firmicutes*, *Bacteroidota* and *Proteobacteria*, but with an overall different proportion among the different strains, and with a relatively similar composition (Figure 2A). At the genus level, the composition was also relatively similar, with *Muribaculaceae*, *Lactobacillus*, and *Bacteroides* being predominant, and it is noteworthy that at the genus level a fraction of the species of each strain was uncultured (Figure 2B). Afterwards, we performed cluster analyses based on microbial species abundance in different strains of mice as well as within strains. The results indicated that samples within different strains were divided into multiple Clades, but most strains (except CO) were dominated by a single Clade, with the remaining Clades accounting for only a relatively small proportion of samples. The strain CO was divided into two distinct Clades within the strain, and the number of samples in these two Clades was similar (Figure 3).

### 3.3 Comparison of Differences in Gut Microbial Abundance in Different Strains of Mice

To further investigate the differences in gut microbiology among different mouse strains, we subjected the data from the four strains to two‐by‐two differential abundance species identification (Supporting Information S1: Figure S1 and Supporting Information S2: Figure S2). We focused on differential abundance species at the genus level, with a maximum of 55 differential species in CO versus KO and a minimum of only 27 differential species in CO versus BO. Concretely, in the CO versus BO grouping, BO was enriched with 15, mainly in *Lachnospiraceae_NK4A136_group* as well as *Alistipes*, etc. and CO was enriched with 12, mainly in *Akkermansia* and *Parasutterella*, etc. In the grouping of BO versus IO, BO was enriched with 23, mainly focused on *Alistipes* and *Alloprevotella*, etc., and IO was enriched with 19, mainly on *Lactobacillus* and *Achromobacter*. In the grouping of BO and KO, BO was enriched with a total of 25, mainly focused on *Lachnospiraceae_NK4A136_group* and *Alistipes*, and KO was enriched with 28, mainly in *Lactobacillus* and *Ralstonia*. In the grouping of CO versus IO, CO was enriched with 21, mainly containing *Akkermansia* and *Alloprevotella*, and IO was enriched with a total of 10, mainly focused on *Lactobacillus* and *Desulfovibrio*. In the grouping of CO and KO, CO was enriched with 28, mainly focused on *Alistipes* and *Lachnospiraceae_NK4A136_group*, KO enriched 27, containing mainly *Lactobacillus* and *Ralstonia*. In the IO versus KO subgroup, IO identified 15, concentrating mainly on *Lachnospiraceae_NK4A136_group* and *Alistipes*, and KO enriched 30, mainly concentrated in *Lactobacillus* and *Ralstonia*. In summary, we found that the presence of *Akkermansia*, *Lactobacillus*, and *Alistipes* in multiple subgroups predicts importance for mouse gut microbes. In addition, it is worth noting that since in the above analysis we found that the CO group could be divided into two distinct Clades within the group, we also compared the abundance differences between these two Clades (Supporting Information S3: Figure S3), and we found that there were a total of 37 differentially abundant species, with the C1 group being enriched mainly for *Escherichia* as well as for *Subdoligranulum*, etc., whereas the C2 group is mainly enriched with *Lachnospiraceae_NK4A136_group* and *Alistipes*, etc.

### 3.4 Characterization of Co‐Abundance Networks in Different Strains of Mice

**Figure 4:**
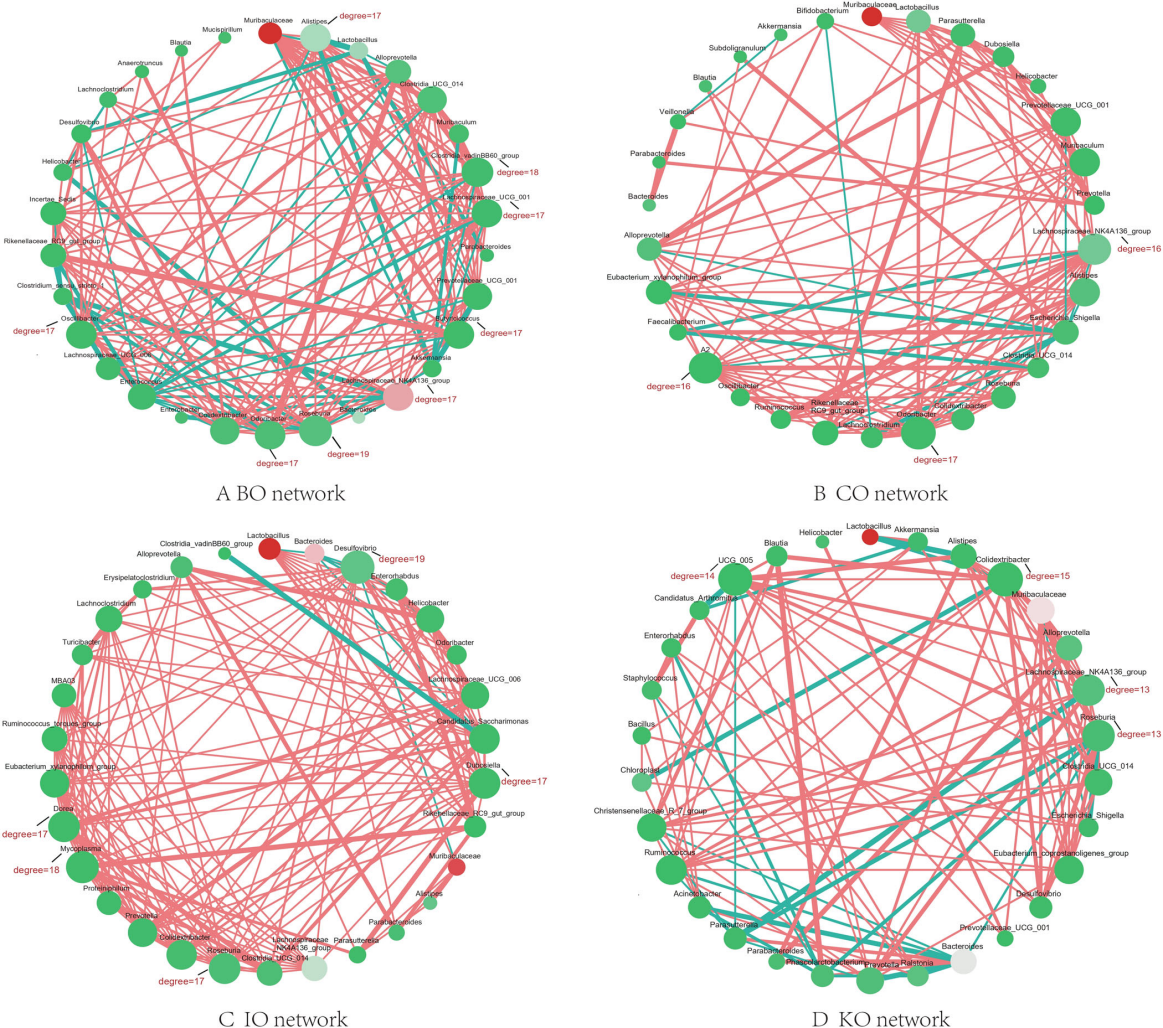
(A) Co‐abundance network of BO mouse. (B) Co‐abundance network of CO mouse. (C) Co‐abundance network of IO mouse. (D) Co‐abundance network of KO mouse.

To study the interactions between bacterial communities in the gut microbiota of different mouse strains, we constructed four co‐abundance networks (Figure 4). The common characteristics of these networks are that the core most nodes all have a degree of around 16–17 and none of them are of high abundance. The networks are mainly dominated by positively correlated connections, while negative correlations are relatively few. Specifically, in BO's network, the core nodes mainly contain two genera of *Clostridia*, *Alistipes*, and *Lachnospiraceae*, and some of the core nodes are also present in the major differential genes mentioned above, and the highest abundance in the network is in a genus of *Muribaculaceae*. In the network of CO, the core nodes mainly contain *Lachnospiraceae_NK4A136_group* as well as *Odoribacter*, and again, the highest abundance of nodes from *Muribaculaceae*. There are several highly connected nodes such as *Desulfovibrio* and *Roseburia* in the IO network and interestingly *Mycoplasma* also has a high degree of connectivity in the network (degree = 18). In the IO network, *Muribaculaceae* remains the most abundant node, in addition to *Lactobacillus*, which also has a high abundance. In the KO network, the core nodes are mainly from *Colidextribacter*, *Lachnospiraceae_NK4A136_group* and *Roseburia*, and the highest abundance in the network is *Lactobacillus*.

### 3.5 Abundances Comparison of Key Gut Microbes in Different Strains of Mice

**Figure 5:**
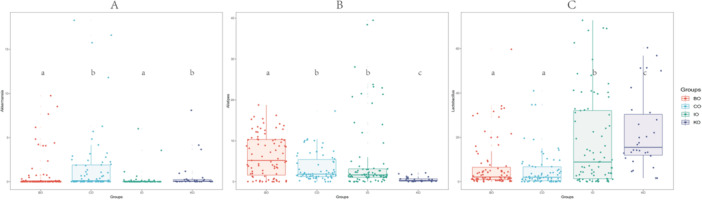
(A) Distribution of genus *Akkermansia*'s abundance of four strains mice. (B). Distribution of genus *Alistipes*'s abundance of four strains mice. (C) Distribution of genus *Lactobacillus*'s abundance of four strains mice.

Based on the above study (Guo et al. 2022; Clavel et al. 2016), we found that some genera recurred in the results, and together with the literature reports, we screened three key genera (i.e., *Akkermansia*, *Alistipes*, and *Lactobacillus*) to characterize their abundance in different strains (Figure 5). Among the four mouse strains, *Lactobacillus* had the highest abundance of gut microbes among the four mouse strains, followed by *Alistipes*, while *Akkermansia* had the lowest abundance. In the comparison of abundance in *Akkermansia*, all three groups except CO had lower abundance and there were significant differences between strains within inbred (CO and BO) and outbred (IO and KO) lines. In the abundance comparison of *Alistipes*, inbred lines demonstrated more abundant than outbred lines, with significant strain differences within each group. In the abundance comparison of *Lactobacillus*, the abundance of the outbred lines was higher than inbred lines. There was a significant difference, with a clear difference within the outbred lines but not within the inbred lines.

## 4 Discussion

Mouse models are extensively utilized in gut microbiology research, serving as pivotal tools for translating basic research findings into clinical applications. However, the impact of gut microbiota differences among various healthy mouse strains on gut microbiology studies has not been systematically evaluated. In this study, we selected the four prevalent mouse strains from inbred (C57BL: CO and BALB: BO) and outbred (KM: KO and ICR: IO) lines to assess gut microbial diversity. To ensure the accuracy of the assessment, we selected public data from multiple studies and retained only the 16S sequencing regions V3‐V4 for the assessment. We hope that this assessment will reveal the background profiles of gut microbes in different mouse strains and the differences in gut microbes between strains, which will contribute to the denoising and reduction of false positives in future mouse gut microbial studies.

In our study, we found that the diversity of inbred lineages was higher than that of outbred lineages, as evidenced by both Shannon and Evenness indices. This may indicate that the microbial communities of outbred lineages are more stable. From the perspective of taxonomic classification, the dominant bacteria exhibited similar patterns at both the phylum and genus levels, with only minor differences in their relative proportions across different lineages. This suggests that the impact of diversity in the microbiota is primarily influenced by the composition of low‐abundance microbiota. We performed cluster analyses within and between strains based on microbial abundance and found that the composition of microbes within strains other than CO was relatively uniform, with only a small number of samples showing bias, suggesting that there was little individual variation in the mice selected within these strains. Notably, two distinct Clusters appeared within the CO strain, and the number of Clusters within these two Clusters was similar, suggesting that one should be mindful of intra‐strain differences when selecting mice from this strain for gut microbiology studies to avoid false positives caused by internal differences. We then performed cluster analysis of all mouse gut microbial samples from the four strains and found that the samples from these strains did not cluster well, so the impact of these differences should be noted in later mouse studies. A similar problem was reflected in the identification of differential microbiota, where we found a number of differential microbiota in the two‐pair identification, which should be labeled for background denoising and reducing false positives in future studies.

While assessing the diversity of mouse gut microbes, we also focused on key species, such as *Akkermansia*, *Lactobacillus*, *Alistipes*, and *Muribaculaceae*, which were recurrent in multiple groups of differentially identified species and interestingly, were also in hub nodes or higher‐abundance species in the co‐abundance network. We selected three species, *Akkermansia*, *Lactobacillus* and *Alistipes*, for detailed comparisons of abundance. *Akkermansia* is an important intestinal probiotic that colonizes the intestinal tracts of humans and other animals, catabolising mucins and using intestinal mucus from the intestinal epithelial surface as a source of carbon, nitrogen, and energy for growth. It plays a pivotal role in maintaining intestinal health, preventing and treating diseases, and in maintaining the health of the intestinal tract. *Lactobacillus* is also a class of probiotic bacteria, widely distributed in the surrounding environment, especially in the digestive tract and vagina, etc. *Lactobacillus* can improve the digestion and absorption of nutrients such as proteins, lactose and calcium, and inhibit the reproduction of spoilage and pathogenic bacteria in the intestinal tract. It is rarely pathogenic, and is considered to be a safe probiotic. *Alistipes* belong to the phylum *Bacteroidetes*, they are specialized anaerobic bacteria that are found in the human gut microbiome and are closely associated with human health and disease. Available research evidence suggests that although they may be associated with disease in some cases, they may also provide protection in others. A comparison of the abundance of the three species revealed that *Lactobacillus* was the most abundant, followed by *Alistipes* and finally *Akkermansia*, and that the abundance of the three species varied in different strains, e.g., the abundance of *Lactobacillus* was higher in the distant lineage than in the inbred lineage, whereas the abundance of *Alistipes* in the inbred lineage was somewhat higher in inbred lines. Clarifying the abundance of these key flora and their roles in the network will help to better understand the gut microbiological background of different strains of mice.

There are some shortcomings in this study: firstly, to ensure the comparability of the data, we restricted factors such as the sequencing method and the amplification region, so we did not use a lot of sample data, which may have some impact on the clustering results of the samples. Secondly, this study mainly focused on 16S sequencing data, so we were unable to differentiate at the species and subspecies level, and we can re‐evaluate the differences in the metagenomic data at a later stage. Finally, although we found differences between strains in terms of species clustering and differential species identification, we are not yet able to assess the impact of these differences on gut microbiology studies or how to select the most suitable mouse model for the study, which may be a concern for us at a later stage.

## 5 Conclusions

In this study, we evaluated the gut microbial profiles of healthy mice from inbred as well as outbred lines based on 16S V3‐V4 sequencing data. Our findings revealed significant differences in the diversity of mouse gut microbes between inbred and outbred strains. In terms of sample clustering, we also found that the clustering of gut microbial samples from the different strains was confusing, and two distinct subgroups also existed within the CO strain. Furthermore, we also identified multiple species with differential abundance between strains, with the abundance of certain key species varying significantly among the four strains. In conclusion, this study suggests that the impact of different strains of gut microbiological background should not be overlooked when using mouse models for gut microbiological studies.

## Author Contributions

**Xudong Liu:** conceptualization, data curation. **Shitao Lian:** methodology, software, data curation. **Aoyi Xiao:** investigation, data curation. **Xiafei Hong:** investigation. **Dingyan Cao:** investigation. **Xinjie Xu:** data curation, investigation. **Yanan Shi:** investigation. **Qing Zhong:** investigation. **Hangqi Liu:** investigation, validation. **Wenjing Wang:** investigation. **Jinyuan Wang:** investigation. **Zilong He:** methodology, data curation, investigation, writing – original draft, writing – review and editing, supervision. **Wenming Wu:** data curation, supervision, investigation, funding acquisition, writing – original draft, writing – review and editing.

## Ethics Statement

The authors have nothing to report.

## Consent

The authors have nothing to report.

## Conflicts of Interest

The authors declare no conflicts of interest.

## Supporting information


**Figure S1.** (A) LEfSe analysis of BO vs. CO. (B) LEfSe analysis of BO vs. IO. (C) LEfSe analysis of BO vs. KO. (D) LEfSe analysis of CO vs. IO. (E) LEfSe analysis of CO vs. KO. (F) LEfSe analysis of IO vs. KO.

**Figure S2.** LEfSe analysis across all four strains.

**Figure S3.** LEfSe analysis of C1 vs. C2.

**Table S1.** Data collection of gut microbiota from different strains of mice.

## Data Availability

Data sharing not applicable to this article as no datasets were generated or analysed during the current study. The dataset analyses during the current study are available in the NCBI database (Supporting Information S4: Table S1).
