# Supplementary figures and images for: Comparison of Gut Microbiological Profiles of Inbred and Outbred Healthy Mice
Source: Microbiologyopen. 2025 Nov 20;14(6):e70134. doi: 10.1002/mbo3.70134 (PMC12631537; doi:10.1002/mbo3.70134)

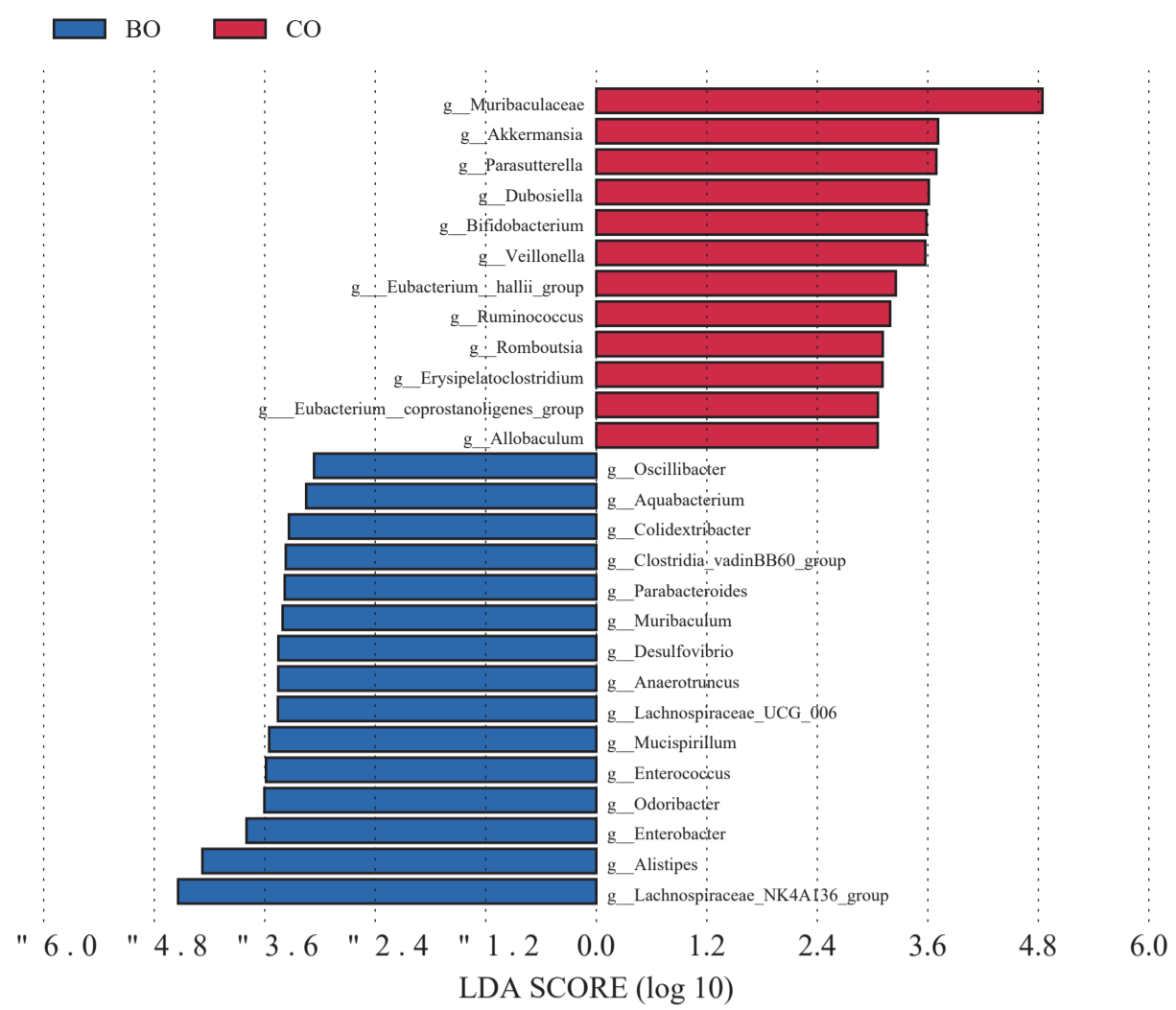

A

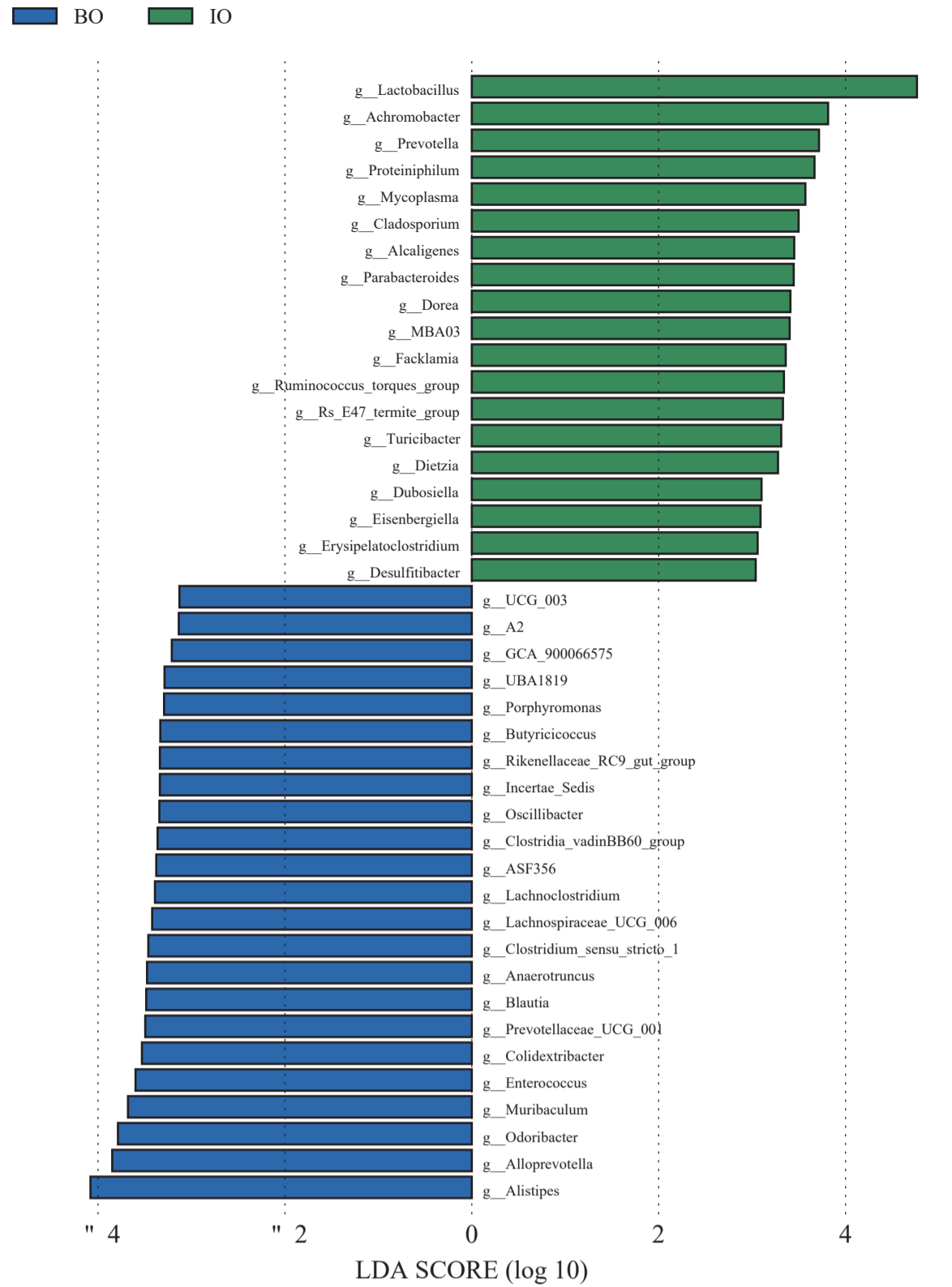

B

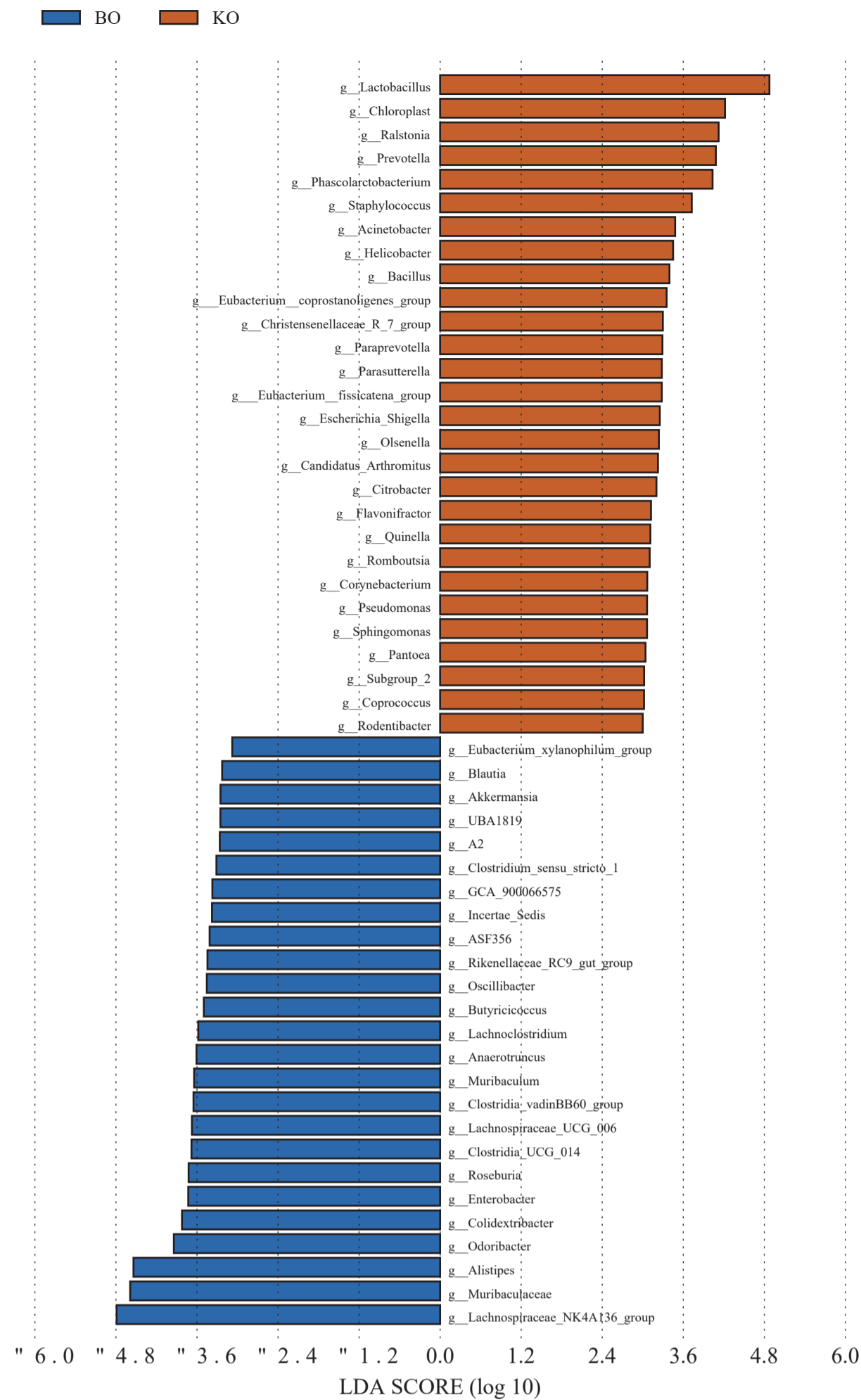

C

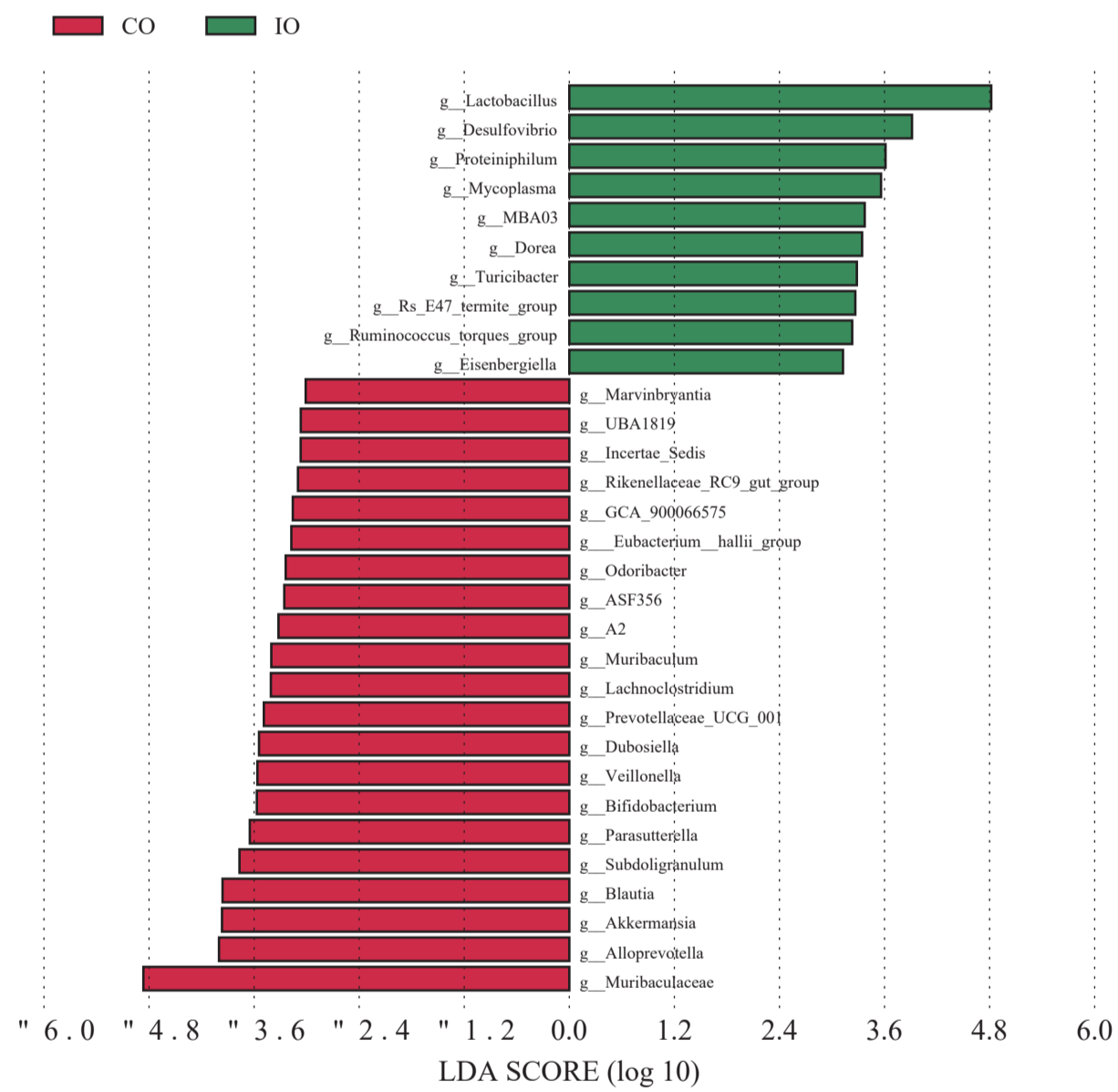

D

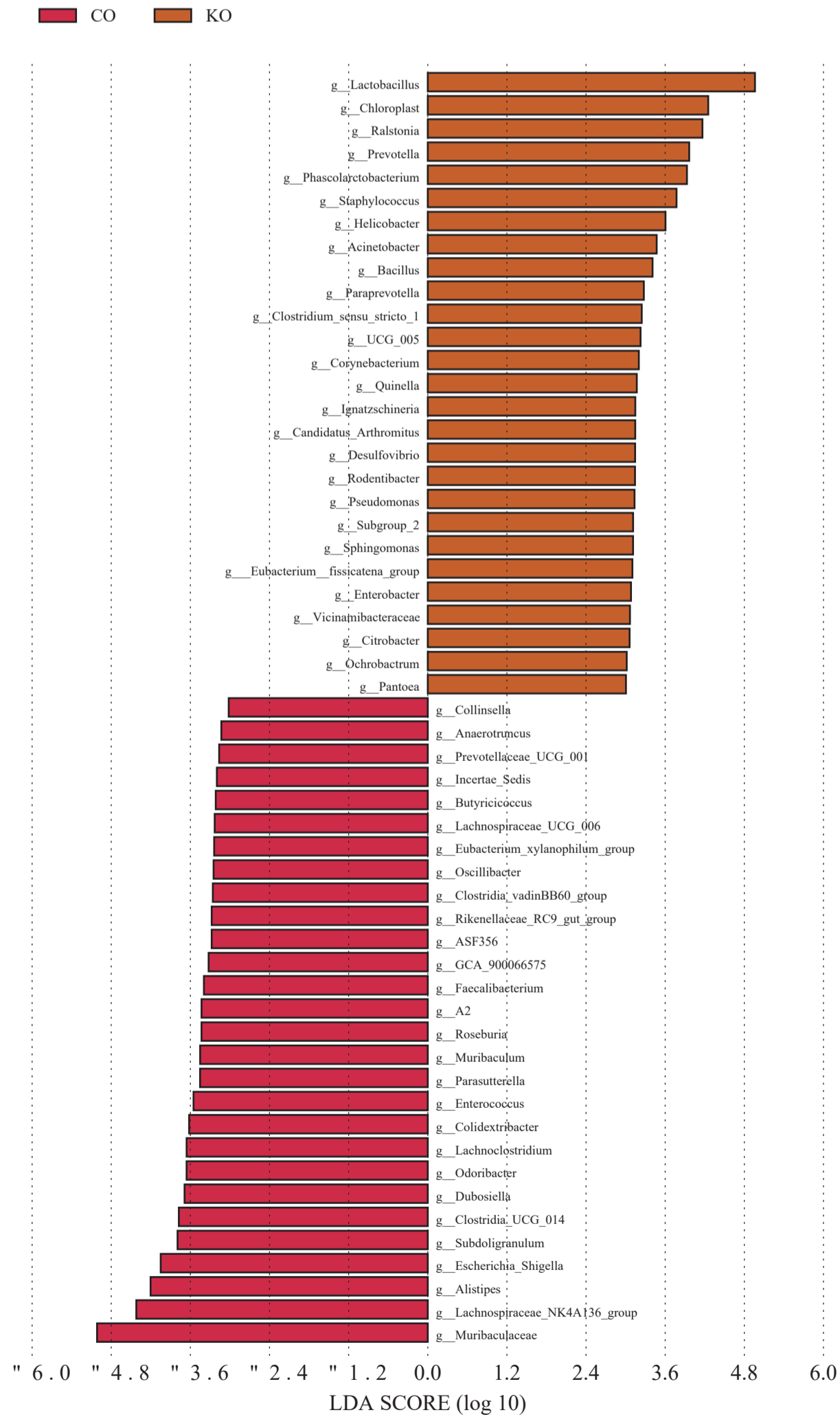

E

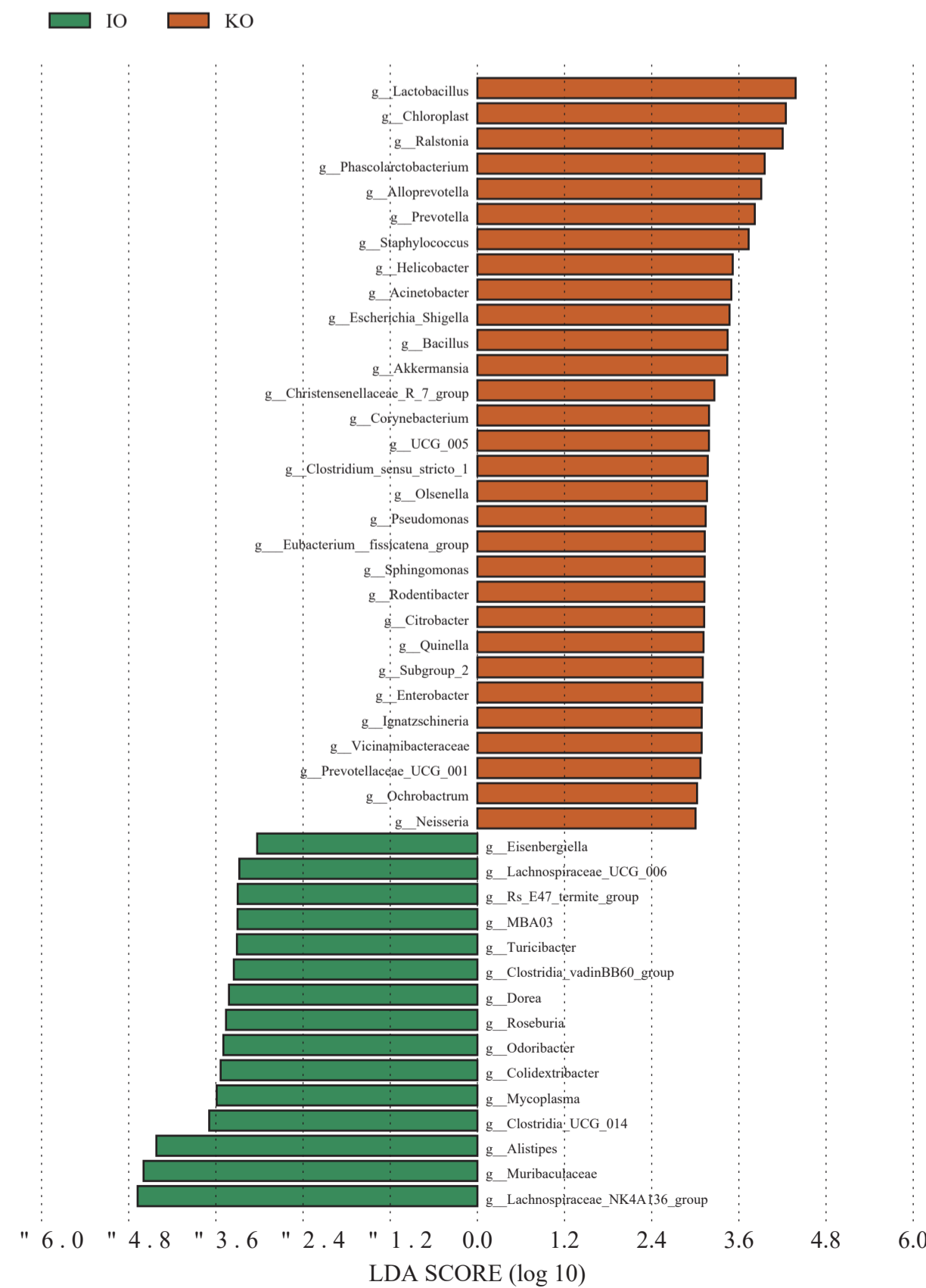

F

Supplement: Supplementary file 1 — Figure S1. (A) LEfSe analysis of BO vs. CO. (B) LEfSe analysis of BO vs. IO. (C) LEfSe analysis of BO vs. KO. (D) LEfSe analysis of CO vs. IO. (E) LEfSe analysis of CO vs. KO. (F) LEfSe analysis of IO vs. KO. [file MBO3-14-e70134-s004.pdf]

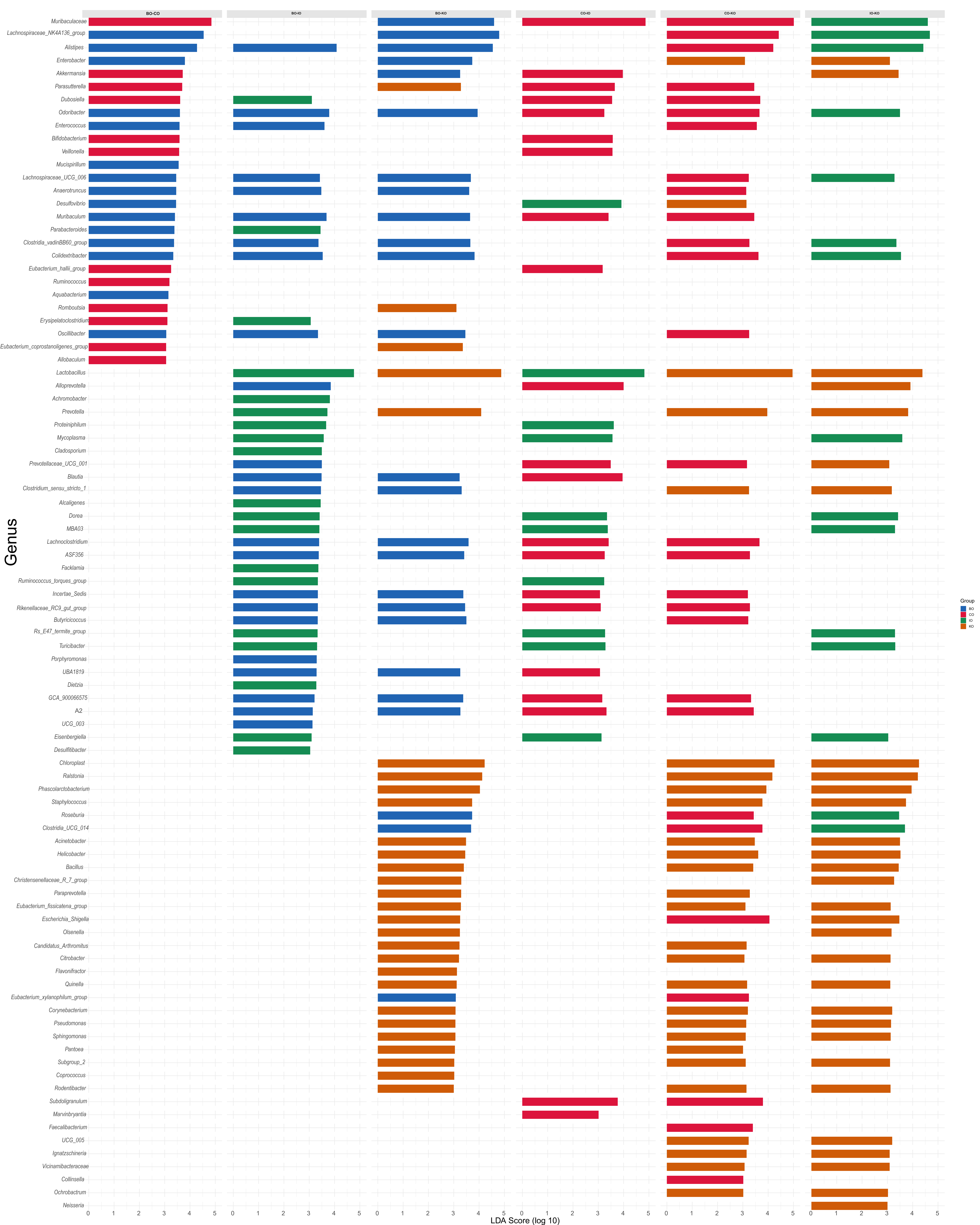

Supplement: Supplementary file 2 — Figure S2. LEfSe analysis across all four strains. [file MBO3-14-e70134-s003.pdf]

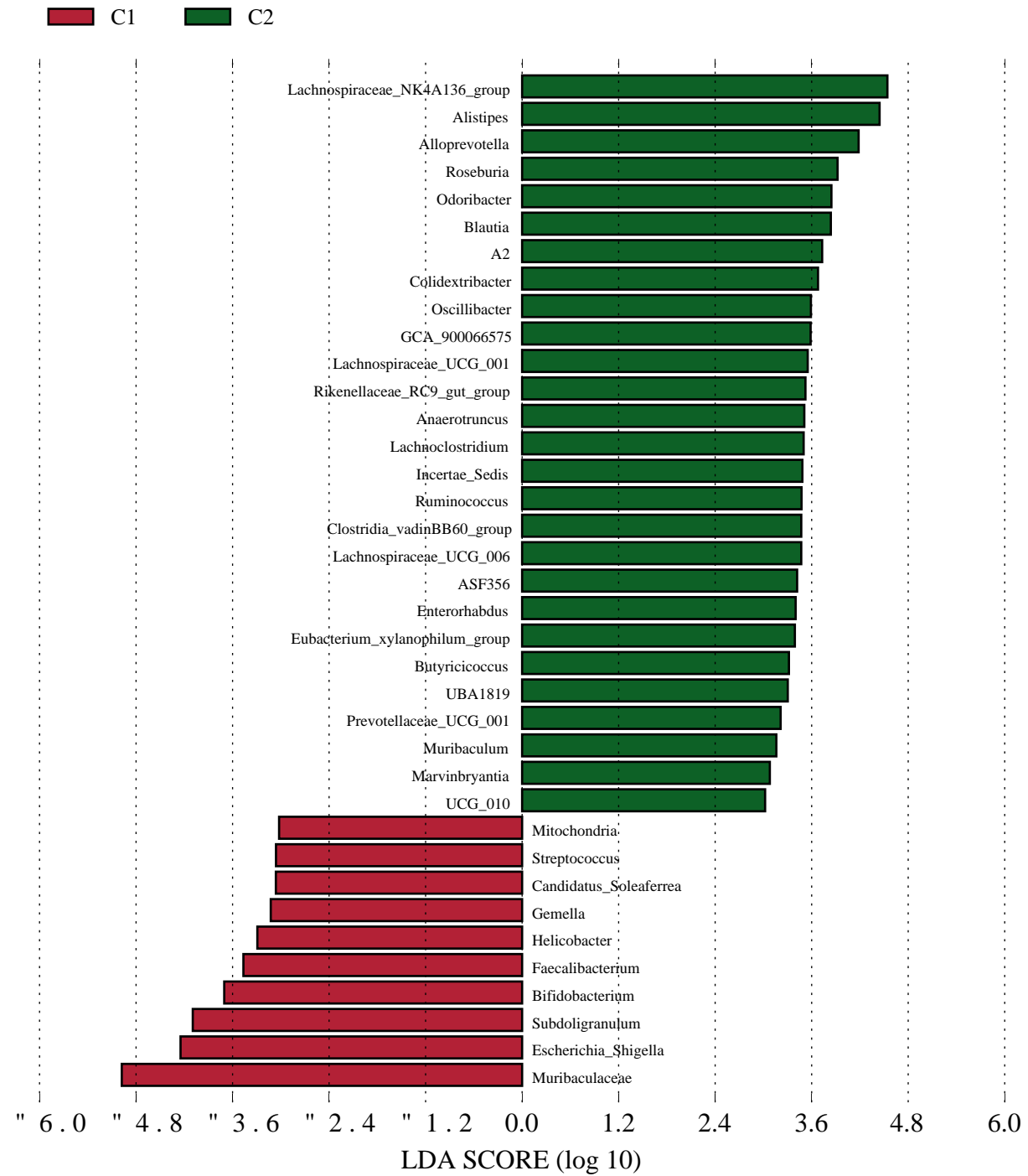

Supplement: Supplementary file 3 — Figure S3. LEfSe analysis of C1 vs. C2. [file MBO3-14-e70134-s002.pdf]
